# Supplementary material for: Initial Step of Selenite Reduction via Thioredoxin for Bacterial Selenoprotein Biosynthesis
Source: Int J Mol Sci. 2021 Oct 11;22(20):10965. doi: 10.3390/ijms222010965 (PMC8538045; doi:10.3390/ijms222010965)
Supplement: Supplementary file 1 [file ijms-22-10965-s001.zip › ijms-1402997-supplementary.pdf]

International Journal of *Molecular Sciences*

MDPI

Article

Initial Step of Selenite Reduction via Thioredoxin for Bacterial Selenoprotein Biosynthesis

Atsuki Shimizu [1,†], Ryuta Tobe [1,†], Riku Aono [1,†], Masao Inoue [1,2], Satoru Hagita [1], Kaito Kiriyama [1], Yosuke Toyotake [3], Takuya Ogawa [3], Tatsuo Kurihara [3], Kei Goto [4], N. Tejo Prakash [5] and Hisaaki Mihara [1,*]

1 College of Life Sciences, Ritsumeikan University, 1-1-1 Nojihigashi, Kusatsu, Shiga 525-8577, Japan; 0229atk33@gmail.com (A.S.); tober0925@gmail.com (R.T.); raono@fc.ritsumei.ac.jp (R.A.); satoru.hagita@daikin.co.jp (S.H.); kirikaiw@gmail.com (K.K.); mihara@fc.ritsumei.ac.jp (H.M.)
2 R-GIRO, Ritsumeikan University, 1-1-1 Nojihigashi, Kusatsu, Shiga 525-8577, Japan; mainoue@fc.ritsumei.ac.jp (M.I.)
3 Institute for Chemical Research, Kyoto University, Gokasho, Uji, Kyoto 611-0011, Japan; toyotake@fc.ritsumei.ac.jp (Y.T.); ogawa.tky@mbc.kuicr.kyoto-u.ac.jp (T.O.); kurihara@scl.kyoto-u.ac.jp (T.K.)
4 Department of Chemistry, School of Science, Tokyo Institute of Technology, 2-12-1 Ookayama, Meguro-ku, Tokyo 152-8551, Japan; goto@chem.titech.ac.jp (K.G.)
5 School of Energy and Environment, Thapar Institute of Engineering and Technology, Patiala, Punjab 147004, India; ntejoprakash@thapar.edu (N.T.P.)
* Correspondence: mihara@fc.ritsumei.ac.jp; Tel.: +81-(0)77-561-2732
† These authors contributed equally to this work.

Supplementary Materials

```
                                                     *           * *  * *  **
Escherichia coli                  1  ----MSDKIIHLTDDSFDTDVLKADGAILVDFWAEWCGPC  36
Pseudomonas stutzeri F2a          1  ----MSDYINNVSDSSFEQDVLQADGPVLVDYWAEWCGPC  36
Nitrosomonas europaea             1  ----MSQHIHYVTDASFESEVLQCPVPVLVDYWAEWCGPC  36
Rhodospirillum rubrum             1  --------MKQVSDASFEEDVLKADGPVLVDFWAEWCGPC  32
Thermodesulfobacterium commune    1  ------MGAPKVTDETFETEVLKSPIPVLVDFWAAWCGPC  34
Helicobacter pylori               1  -----MSHYIELTEENFESTI--KKGVALVDFWAPWCGPC  33
Thermus thermophilus              1  -----MAKPIEVTDQNFDETLGQHP-LVLVDFWAEWCAPC  34
Prochlorococcus marinus           1  -----MSSAAAVTDSSFEQEVLQSDLPVLVDFWAPWCGPC  35
Mycobacterium tuberculosis        1  MTDSEKSATIKVTDASFATDVLSSNKPVLVDFWATWCGPC  40
Chlamydia trachomatis             1  -------MVQVVSQENFADSI--ASGLVLIDFFAEWCGPC  31
Bacillus subtilis                 1  ------MAIVKATDQSFSAET--SEGVVLADFWAPWCGPC  32

                                         *                *   *             *
Escherichia coli                 37  KMIAPILDEIADEYQGKLTVAKLNIDQNPGTAPKYGIRGI  76
Pseudomonas stutzeri F2a         37  KMIAPVLDEIAKDYEGRLKVCKLNIDENQETPPKYGVRGI  76
Nitrosomonas europaea            37  RMIAPLLDEIASEYGDRLKIAKLNIDENQSTPQKYGIRGI  76
Rhodospirillum rubrum            33  RQIAPALEELATALGDKVTVAKINIDENPQTPSKYGVRGI  72
Thermodesulfobacterium commune   35  RVIAPIIDELAEEFEGKVKVMKLNVDENPVTPGKYGIRAI  74
Helicobacter pylori              34  KMLSPVIDELASEYEGKAKICKVNTDEQEELSAKFGIRSI  73
Thermus thermophilus             35  RMIAPILEEIAKEYEGKLLVAKLDVDENPKTAMRYRVMSI  74
Prochlorococcus marinus          36  RMVSPIVDEISKDFEGKIKVCKLNTDENPNVASQYGIRSI  75
Mycobacterium tuberculosis       41  KMVAPVLEEIATERATDLTVAKLDVDTNPETARNFQVVSI  80
Chlamydia trachomatis            32  KMLTPVLEALAAELP-HVTILKVDIDSSPRPAEQYSVSSI  70
Bacillus subtilis                33  KMIAPVLEELDQEMGDKLKIVKIDVDENQETAGKYGVMSI  72

                                     **    * *       *   *
Escherichia coli                 77  PTLLLFKNGEVAATKVGALSKGQLKEFLDANLA---      109
Pseudomonas stutzeri F2a         77  PTLMLFKNGNVEATKVGALSKSQLAAFLDSNI----      108
Nitrosomonas europaea            77  PTLMIFKNGNIEATKVGALSKSQLTAFVDSHL----      108
Rhodospirillum rubrum            73  PTLMIFKDGQVAATKIGALPKTKLFEWVEASV----      104
Thermodesulfobacterium commune   75  PTLIIFKNGEPVEVIVGAVSKNTIVNALNKVLA---      107
Helicobacter pylori              74  PTLLFTKDGEVVHQLVGVQTKVALKEQLNKLLG---      106
Thermus thermophilus             75  PTVILFKDGQPVEVLVGAQPKRNYQAKIEKHLPATA      110
Prochlorococcus marinus          76  PTLMIFKGGQKVDTVVGAVPKATLSGTISKHL----      107
Mycobacterium tuberculosis       81  PTLILFKDGQPVKRIVGAKGKAALLRELSDVVPNLN      116
Chlamydia trachomatis            71  PTLILFKDGKEVERSVGLKDKDSLIKLISKHQ----      102
Bacillus subtilis                73  PTLLVLKDGEVVETSVGFKPKEALQELVNKHL----      104
```

Figure S1. Amino acid sequence alignment of thioredoxin homologs from various bacterial phyla. Sequences were aligned using Muscle [1]. The amino acid sequences used in this alignment were obtained from protein databases with locus tags of b3781

(*Escherichia coli*), PszF2a_05700 (*Pseudomonas stutzeri* F2a), NE1034 (*Nitrosomonas europaea*), Rru_A3433 (*Rhodospirillum rubrum*), HL41_06235 (*Thermodesulfobacterium commune*), HP_0824 (*Helicobacter pylori*), TT_C1385 (*Thermus thermophilus*), Pro_1139 (*Prochlorococcus marinus*), Rv3914 (*Mycobacterium tuberculosis*), CT_539 (*Chlamydia trachomatis*), and BSU28500 (*Bacillus subtilis*). Yellow highlight, catalytic Cys residues. Asterisks, conserved residues.

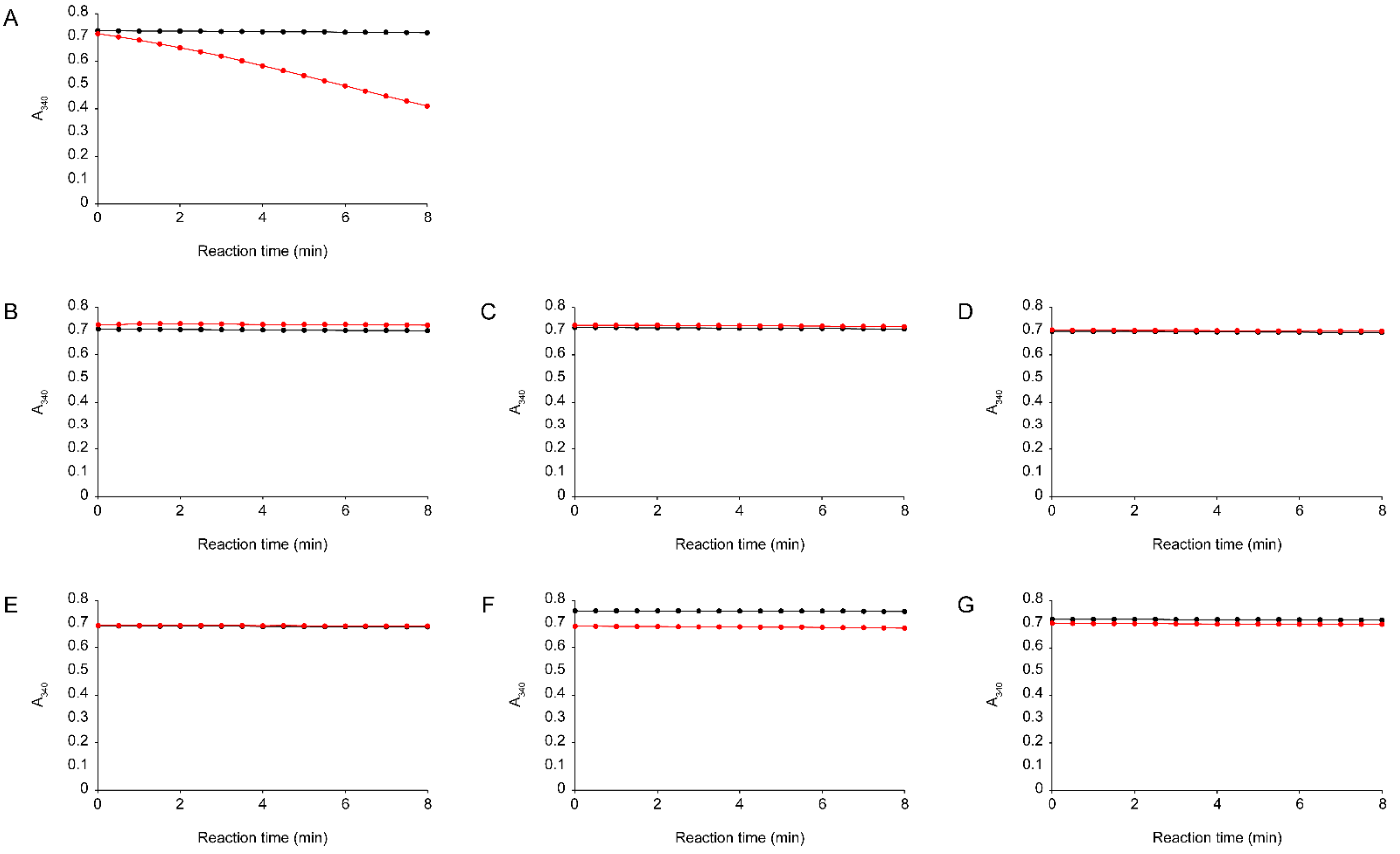

Figure S2. Oxyanion reduction activity of Trx system from *P. stutzeri* F2a. The activity towards selenite (A), selenate (B), sulfite (C), sulfate (D), thiosulfate (E), nitrite (F), and nitrate (G) was measured using PsTrxA with PsTrxR and NADPH by monitoring the decrease in A_{340} due to NADPH oxidation. The assay was performed with (red) or without (black) PsTrxA.

Table S1. Primers used in this study

Primer names	Primer sequences
PsTrxA-f	5'-GGGAATTCCATATGAGCGACTACATCAACAATGTCAGCG-3'
PsTrxA-r	5'-CGCGGATCCTCAGATGTTGCTGTCGAGGAAGGC-3'
PsTrxR-f	5'-GGGAATTCCATATGAGTGAAGTCAAGCATTCGCGTCTG-3'
PsTrxR-r	5'-CGCGGATCCTCAATTGTCCAGGTACTTCTCGGCATC-3'
PsTrxA_C33A-f	5'-ACTGGGCTGAGTGGGCTGGCCCCTGCAAGA-3'
PsTrxA_C33A-r	5'-TCTTGCAGGGGCCAGCCCACTCAGCCCAGT-3'
PsTrxA_C36A-f	5'-AGTGGTGTGGCCCCGCCAAGATGATCGCGC-3'
PsTrxA_C36A-r	5'-GCGCGATCATCTTGGCGGGGCCACACCACT-3'
PsTrxA_C33A/C36A-f	5'-ACTGGGCTGAGTGGGCTGGCCCCGCCAAGA-3'
PsTrxA_C33A/C36A-r	5'-TCTTGGCGGGGCCAGCCCACTCAGCCCAGT-3'

References

1. Edgar, R. C., MUSCLE: multiple sequence alignment with high accuracy and high throughput. *Nucleic Acids Res.* **2004,** 32, (5), 1792-7.
